# Successful pregnancy in a complex chromosomal rearrangement carrier using preimplantation genetic testing for structural rearrangements: A case report

**DOI:** 10.1097/MD.0000000000041327

**Published:** 2025-01-31

**Authors:** Yacong Wang, Xue Ke, Xuefei Liang, Xiaolan Ouyang, Xiaoxi Yang, Fang Wang

**Affiliations:** a Department of Reproduction and Infertility, Chengdu Women’s and Children’s Central Hospital, School of Medicine, University of Electronic Science and Technology of China, Chengdu, China.

**Keywords:** blastocyst, intracytoplasmic sperm injection, karyotypes, reciprocal translocation, Robertson translocation

## Abstract

**Rationale:**

Complex chromosomal rearrangements (CCRs) frequently lead to unfavorable reproductive consequences, such as recurrent miscarriage. Preimplantation genetic testing for structural rearrangements (PGT-SR) has become a successful method for embryo diagnosis in CCR families.

**Patient concerns:**

A woman of childbearing age experienced repeated miscarriages during her attempts to conceive.

**Diagnoses:**

A woman was diagnosed with a combined Robertsonian translocation involving chromosomes 13 and 15 (robs[13;15]) and a reciprocal translocation involving chromosomes 10 and 12 (t[10;12]). She had undergone 5 previous embryonic terminations.

**Interventions:**

The couple underwent assisted reproductive technology with ovulation induction using a high-progesterone progestin-primed ovarian stimulation protocol, followed by intracytoplasmic sperm injection and blastocyst culture. Embryo biopsy was carried out on days 5 and 6, and PGT-SR was employed for genetic diagnosis.

**Outcomes:**

After aneuploidy detection by PGT-SR, the sole remaining blastocyst underwent preimplantation genetic testing for aneuploidy for confirmation and subsequent transfer. Prenatal diagnosis and follow-up after birth were conducted.

**Lessons:**

For CCR carriers, particularly couples with a history of recurrent abortion, PGT-SR has the potential to address their reproductive predicament by enhancing their likelihood of achieving a successful pregnancy.

## 1. Introduction

Balanced chromosomal rearrangements, including reciprocal translocations, inversions, and Robertson translocations, are the most common structural chromosomal abnormalities in the general population. While balanced chromosomal rearrangements typically do not cause health problems, they can segregate in either a balanced or unbalanced manner.^[1]^ The balanced form rarely develops a clinical phenotype unless key genes are disrupted at breakpoints.^[2]^ However, the unbalanced manner may result in infertility, recurrent miscarriages, or offspring with developmental delays, intellectual disabilities, and congenital disabilities.^[3]^ The most common balanced translocations are Robertsonian and reciprocal. Robertsonian translocations involve the breakage and fusion of 2 acrocentric chromosomes, whereas reciprocal translocations involve the exchange of distal segments between 2 non-acrocentric chromosomes. Carriers of Robertsonian translocations have an increased risk of infertility, spontaneous abortions, or chromosomally unbalanced offspring.^[4]^ For reciprocal translocations, the likelihood of miscarriage is influenced by the size of the rearranged chromosomal segments and the amount of genetic material involved.^[5]^

Complex chromosomal rearrangements (CCRs) are rare structural rearrangements involving 3 or more cytogenetic breakpoints on more than 2 chromosomes.^[6]^ CCRs can be familial or denovo.^[7]^ CCRs are classified according to the number of chromosomes involved, the number of chromosome breaks, and the involvement of intrachromosomal or interchromosomal insertions. CCRs are usually classified as 3-way rearrangements, double 2-way translocations, or exceptional CCRs.^[8]^ The potential risk of chromosome imbalance in the gametes of CCR carriers is higher than that in those with simple translocations, thus contributing to a higher risk of recurrent miscarriage.^[9]^ According to previous studies, the incidence of spontaneous abortions and abnormal pregnancy outcomes in families with CCR was estimated to be 48.3% and 53.7%, respectively.^[10]^ The number of chromosomes and breakpoints involved in CCRs leads to a wide variety of possible gametes.^[11]^ The odds of balanced or normal embryos in couples with balanced CCRs were very low (<6%).^[12]^ Moreover, female CCR carriers had a lower proportion of normal/balanced embryos (4.9%) compared with male carriers (19.4%).^[6]^

Chromosome rearrangement detection before implantation (i.e., preimplantation genetic testing for structural rearrangement, PGT-SR) is an embryo genetic testing technology that detects embryonic chromosomes using whole genome amplification (WGA) and sequencing technology to identify euploid embryos for transplantation, thus significantly aiding couples with abnormal chromosome structures to obtain clinical pregnancy. In recent years, many centers worldwide have successfully utilized PGT-SR to achieve embryonic diagnosis in families with CCR.^[11,13,14]^

In this case, we successfully blocked CCRs using preimplantation genetic diagnosis (PGD), thereby aiding families with CCR to conceive healthy babies by blocking the transmission of Robertsonian translocation to the offspring. We hope these experiences provide a valuable reference for families with similar CCRs considering assisted reproductive technologies.

## 2. Case presentation

**Figure 1.**
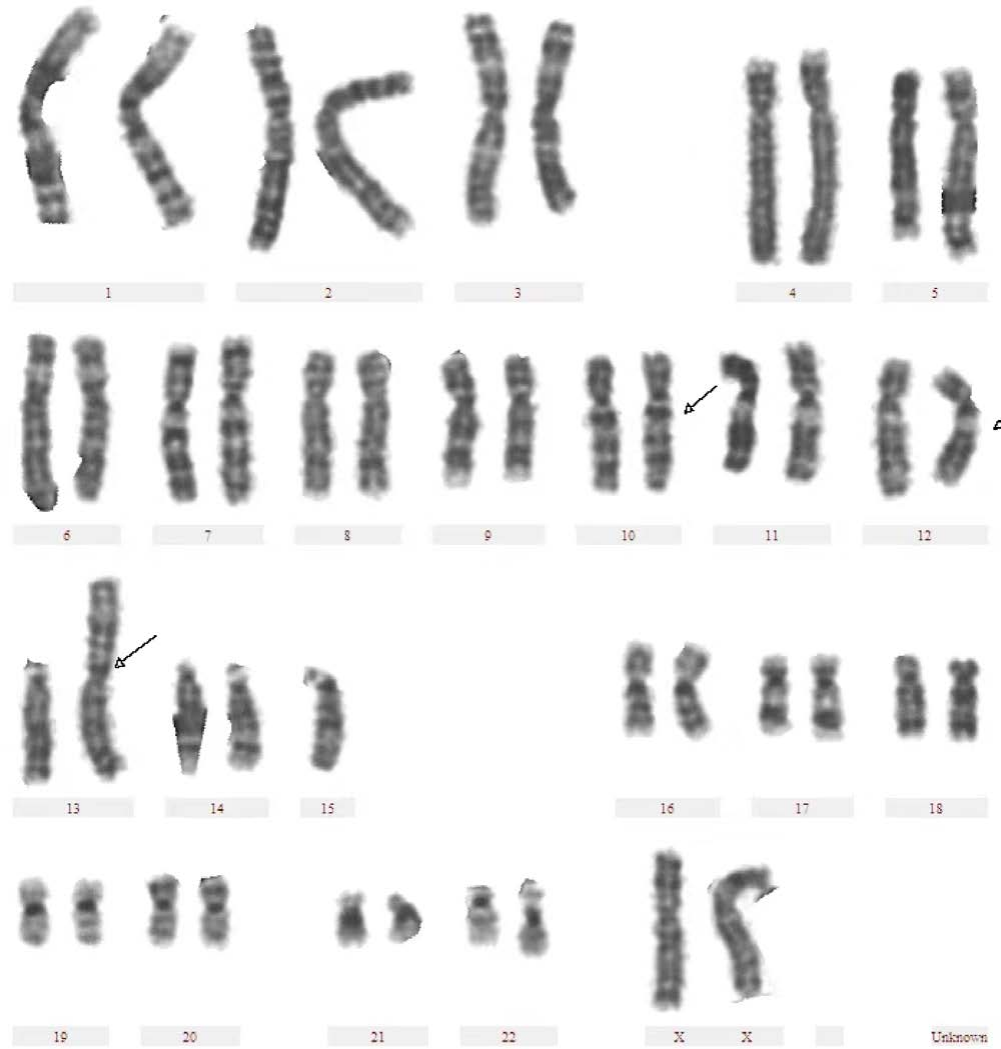
Chromosome karyotype of the female patient, which is 45, XX, *t* (10; 12) (q26; q21.1), der (13; 15) (q10; q10).

A 34-year-old female patient presented with a history of 5 prior pregnancies (last pregnancy in December 2018), all of which resulted in a miscarriage at approximately 2 months’ gestation. Genetic testing was not performed on miscarriage tissues. In March 2015, the patient underwent peripheral chromosome karyotype testing (white blood cell count) at an external hospital. The female’s chromosome karyotypes were 45, XX, *t* (10; 12) (q26; q21.1), der (13; 15) (q10; q10) (Fig. 1). The male partner has a chromosome karyotype of 46, XY. The female partner had normal phenotype and intelligence, no abnormalities in external genitalia, regular menstruation, and normal ovarian function. After genetic counseling, the couple opted for a PGT-SR-assisted pregnancy.

### 2.1. Embryo acquisition and biopsy

Based on the patient’s baseline serum hormone levels, bilateral ovarian antral follicle count via vaginal ultrasound, and body mass index (BMI), an appropriate ovulation induction regimen was selected. The patient underwent 1 cycle of controlled ovulation induction using a high-progesterone progestin-primed ovarian stimulation protocol for 9 days. When at least 2 dominant follicles reached a diameter exceeding 18 mm, ovulation was triggered with an intramuscular injection of 8000 to 10,000 IU human chorionic gonadotropin (HCG). After injecting HCG for 34 to 36 hours, transvaginal ultrasonography and puncture were performed to retrieve the oocytes.

After collecting the oocytes, intracytoplasmic sperm injection was used for fertilization. The embryos were then cultured in vitro until the blastocyst stage, at which point a blastocyst biopsy was performed. Five to ten trophectoderm cells obtained from the biopsy, along with peripheral blood samples from both partners, were sent for PGT-SR analysis. The biopsied embryos were cryopreserved for subsequent transfer.

### 2.2. PGT-SR testing

Trophoblast cell samples underwent a series of multiple annealing and looping-based amplification cycles utilizing a commercially available single-cell WGA kit (REPLI-g^®^ Single Cell Kit, Germany). Genomic DNA (gDNA) was extracted from the couple’s peripheral blood leukocytes using a TIANamp Blood DNA kit (TIANGEN, Beijing, China). WGA products and genomic DNA samples were loaded onto Illumina BeadChips (San Diego) for single nucleotide polymorphism (SNP) analysis using the iScan Control Software system and iScan scanner (Illumina). The resulting SNP data was used to analyze the integrity of 23 pairs of chromosomes in the embryos and the SNP haplotype carried by the family. Furthermore, high-throughput next-generation sequencing was performed on the DA8600 sequencing platform (Basecare Medical Device Co., Ltd., Jiangsu, China) to confirm embryo ploidy status (euploid or aneuploid).

### 2.3. CNVs analysis and SNP haplotype analysis

Copy number variations (CNVs) in the embryos were analyzed using a combined approach of B-allele frequency (BAF) and Log R ratio (LRR) values. BAF refers to the proportion of B alleles detected by the Illumina Infinium genotyping platform. In normal samples, BAF values for AA, BB, and AB typically approach 0.1 and 0.5, respectively. If the BAF value of a region is close to 0.33 or 0.66, duplication may occur in this region (AAB, ABB). Additionally, if the BAF value of a segment is 0 or 1, this indicates that a deletion or region of homozygosity (ROH) may occur. LRR values were used for further confirmation in these cases. An LRR value of 0 suggests the presence of 2 copies (normal state), whereas an LRR value <0 indicates a deletion. Based on the proportion of cells with detected CNVs, blastocysts were classified as euploid (<30% abnormal cells), mosaic (30–70% abnormal cells), or aneuploid (>70% abnormal cells).

To establish the family’s SNP haplotype, informative SNP sites were identified based on parental genotypes (e.g., father AA and mother AB, where B represents the mother’s informative locus). The presence of the B allele in an embryo indicated maternal origin. Since no samples from offspring with chromosomal imbalances were available, embryos generated from a 2:2 segregation of adjacent chromosomal positions were used as standard reference embryos. Finally, the embryos were classified based on SNP haplotypes to distinguish between embryos carrying the chromosomal rearrangements and those with normal karyotypes.

## 3. Clinical findings and outcomes

**Figure 2.**
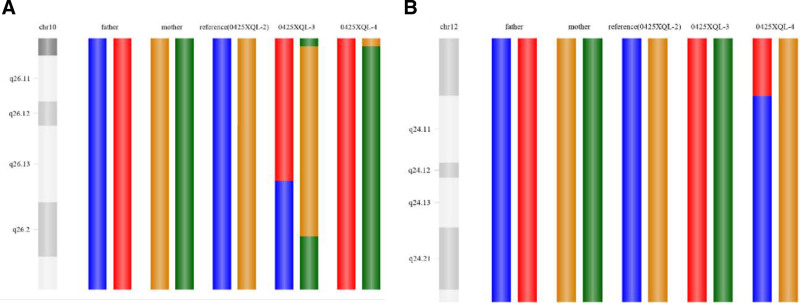
SNP haplotypes analysis of the *t* (10; 12) (q26; q21.1). (A) SNP haplotype analysis of breakpoint on chromosome 10. (B) SNP haplotype analysis of breakpoint on chromosome 12. SNP = single nucleotide polymorphism.

The patients with CCR underwent a single cycle of PGT-SR. Six oocytes were retrieved, and 3 blastocysts on Day 5 (D5) and 1 blastocyst on Day 6 (D6) were obtained following intracytoplasmic sperm injection insemination. Biopsy samples from all 4 blastocysts were analyzed using PGT-SR. SNP haplotype construction for der (10; 12) (q26; q21.1) was successful (Fig. 2). However, owing to the lack of reference embryos with the der (13; 15) (q10; q10) translocation, SNP haplotype construction for this rearrangement was not possible. The results of the PGT-SR tests are shown in Table 1.

**Table 1:** PGT-SR results.

Embryo number	Embryonic morphological grading	SNP haplotypes [*t* (10; 12) (q26; q21.1)]	CNVs
1	4BB	Imbalanced translocation type	del(10)(q26.13q26.3); dup(12)(q24.12q24.33); del(mosaic)(3)(q12.1q29)(30%)
2	4BB	Imbalanced translocation type	del(9)(q21.11q34.3); dup(10)(q26.13q26.3); del(12)(q24.12q24.33); del(mosaic)(1)(q31.1q44)(30%)
3	4BC	Normal type	dup (7) (q11.23)(2.38Mb)
4	5BC	Translocation-carrying type	Euploid

CNV = copy number variation; PGT-SR = preimplantation genetic testing for structural rearrangements, SNP = single nucleotide polymorphism.

**Figure 3.**
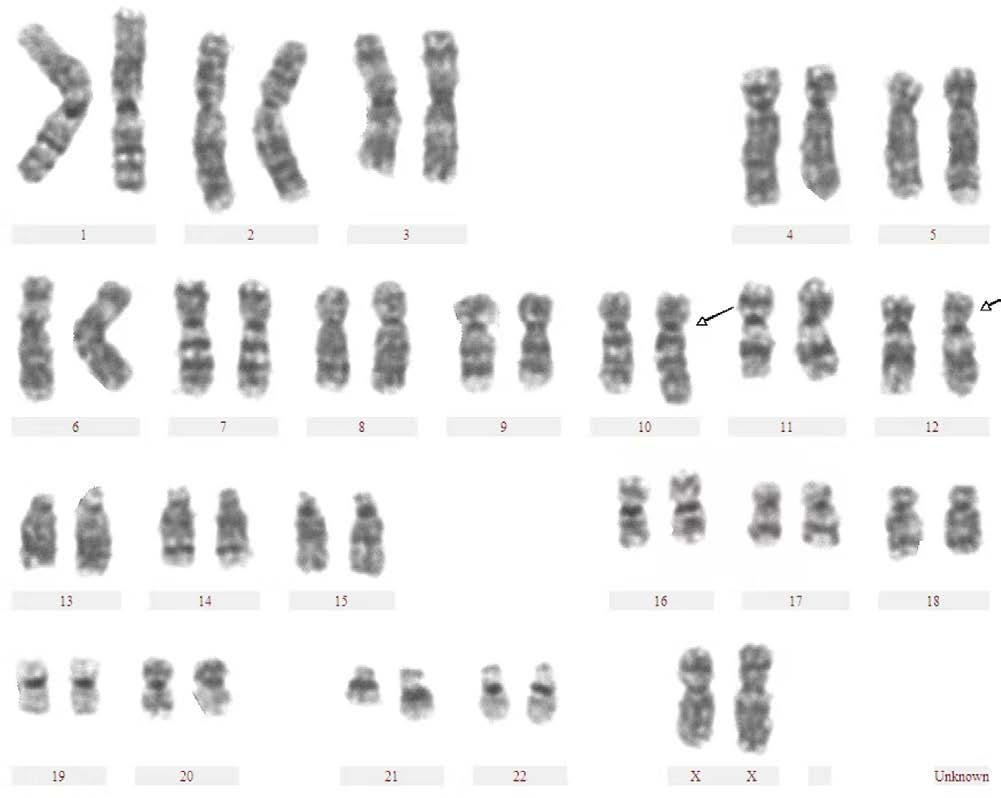
Chromosome karyotype of amniotic fluid sample, which is 46, --, *t* (10; 12) (q26; q24.1).

Following detailed genetic counseling, the couple opted for the transfer of a single blastocyst. A clinical pregnancy was achieved on November 19, 2022, through artificial freeze-thaw embryo transfer. At 18 weeks of pregnancy, amniocentesis was performed for prenatal diagnosis. The amniotic fluid cell chromosome karyotype was 46-- XX, *t* (10; 12) (q26; q24.1) (Fig. 3), and the SNP chip did not detect clinically significant chromosomal CNVs. On August 9, 2023, a healthy female infant weighing 3.7 kg and measuring 48 cm in length was delivered at full term.

## 4. Discussion

CCR carriers generally have a normal phenotype; however, they can experience various reproductive challenges, including infertility, recurrent miscarriages, and offspring with congenital malformations.^[15]^ Female carriers usually have recurrent spontaneous abortion. We found an early case in a family in India that showed both types of translocation in the same patient, rob (13;14) and *t* (4;7), with a history of recurrent abortions.^[16]^ The presente case describes a woman diagnosed with a combined CCR involving a Robertsonian translocation (rob[13;15]) and a reciprocal translocation (*t* [10;12]). The couple visited our center after 5 repeated spontaneous abortions, and their clinical symptoms align with the known reproductive complications associated with CCRs. For couples who have experienced spontaneous abortion, attention should be paid to the detection of chromosome karyotype and identify chromosomal abnormalities quickly to give fertility guidance, avoid the risk of experiencing multiple abortions, and mitigate physical and psychological harm.

As early as 2001, Durban et al^[17]^ utilized PGT technology to identify female carriers of balanced Robertsonian and reciprocal translocations using first polar body (1PB) analysis.^[17]^ They successfully used 1PB biopsy and PGT technology in 6 clinical cases (4 Robertsonian translocations and 2 reciprocal translocations). Nowadays, a 1PB biopsy is no longer the mainstream biopsy method used for PGD. Currently, blastocysts cultured for 5 to 6 days are the preferred biopsy material used in PGD due to their ease of operation and standardization. Our study employed this current standard approach. It is extremely difficult for this CCRs carrier to generate a normal/balanced gamete. As a result, it has a higher risk of spontaneous miscarriage or chromosomal abnormalities.

Batista et al^[18]^ analysis of cases of CCRs and review of familial cases middle finger. The risk of spontaneous abortion in CCRs carriers can reach 50%~100%. Gorski et al^[10]^ estimated the general risk of spontaneous abortion to be 48.3% and the general risk of abnormal children to be 18.4%, based on 67 pregnancies in 25 families (mostly 3-way rearranged CCRs), but this does not mean that patients with CCRs will absolutely lose the opportunity to have their own children. Most CCRs are unique within each carrier or family, and different CCRs should be individualized.^[19]^

As new technologies in the field of assisted reproduction continue to emerge, there are more opportunities for CCRs carriers. PGT has been suggested as an adjunctive treatment strategy for fertility problems in CCRs carriers. Lim et al^[20]^ reported 3 patients in the process, a healthy live birth was obtained after 4 cycles of PGT. Li et al^[21]^ reported that 2 patients had successfully delivered live births after 3 PGT cycles, and 1 healthy male infant carrier of CCRs was born. This case showed a successful outcome despite the female partner’s age (34 years) being close to the advanced maternal age range and the female carrying a more complex CCR. However, only a limited number of oocytes (6) were retrieved. We screened out 1 chromosome-balanced blastocyst from 4 blastocysts in just 1 PGT-SR cycle and obtained a healthy baby after embryo transfer.

The early follicular long-acting gonadotropin-releasing hormone Agonist Long Regimen (EFLL) is a popular controlled ovarian hyperstimulation regimen widely used in China. A large-scale study^[22]^ from China found that compared with the medium luteal short-acting gonadotropin-releasing hormone agonist long regimen regimen, the use of the EFLL regimen in PGT-SR cycles resulted in more diploid embryos. Based on this theoretical assumption, if the EFLL scheme is used in women, then more diploid embryos may be obtained. However, this must be verified in future studies. In this case, the controlled ovulation induction regimen used was progestin-primed ovarian stimulation, which was considered to reduce the cost of treatment. Due to the need to wait for the results of embryo biopsy testing, PGT usually does not require fresh embryo transfer, therefore, endometrial receptivity is not the primary consideration.

This case report contributes significantly to the existing literature on PGT-SR for CCRs. While previous studies have established the effectiveness of PGT-SR in overcoming infertility in CCR carriers, our case report provides the first successful pregnancy outcome for a woman with a combined Robertsonian translocation and reciprocal translocation. Furthermore, the success was achieved despite the woman’s age and limited oocytes, suggesting that these factors may not be as influential on PGT-SR outcomes as previously thought. This finding has potential implications for expanding access to PGT-SR for a wider range of CCR carriers. Additionally, the study highlights the importance of comprehensive PGT-SR analysis, as evidenced by the identification of a chromosomal abnormality in 1 blastocyst lacking the parental translocations.

## 5. Conclusion

PGT-SR technology can help CCR carrier couples seeking chromosomally balanced children. In this case, an older couple with CCRs achieved a successful pregnancy and delivery of a healthy baby using PGT-SR technology to identify and transfer a single euploid embryo. This case further reinforces the potential of PGT-SR in overcoming the reproductive challenges faced by CCR carriers, particularly couples with a history of recurrent miscarriage. The PGT-SR technique can significantly reduce the occurrence of adverse pregnancy outcomes due to chromosomal structural abnormalities in couples, which is a better choice for such families to achieve normal fertility.

## Acknowledgments

The authors thank the staffs of the Department of Chengdu Women’s and Children’s Central Hospital for their cooperation and support.

## Author contributions

**Data curation:** Xiaolan Ouyang.

**Funding acquisition:** Xue Ke.

**Methodology:** Xiaolan Ouyang.

**Resources:** Xuefei Liang.

**Supervision:** Fang Wang.

**Visualization:** Xiaoxi Yang.

**Writing – original draft:** Yacong Wang.

**Writing – review & editing:** Xue Ke.
